# *Xanthomonas euvesicatoria*-Specific Bacteriophage BsXeu269p/3 Reduces the Spread of Bacterial Spot Disease in Pepper Plants

**DOI:** 10.3390/plants12193348

**Published:** 2023-09-22

**Authors:** Elena Shopova, Liliana Brankova, Sergei Ivanov, Zoltan Urshev, Lyudmila Dimitrova, Melani Dimitrova, Petya Hristova, Yoana Kizheva

**Affiliations:** 1 Institute of Plant Physiology and Genetics, Bulgarian Academy of Sciences, Acad. G. Bonchev Str., bl. 21., 1113 Sofia, Bulgaria; kostei@abv.bg (E.S.); dim.lyudmila@gmail.com (L.D.); 2 Centre of Food Biology, 1592 Sofia, Bulgaria; sivanov714@abv.bg; 3 Faculty of Biology, Sofia University St. “Kliment Ohridski”, 8 Dragan Tzankov Blvd., 1164 Sofia, Bulgaria; mreftimova@uni-sofia.bg (M.D.); pkabad@biofac.uni-sofia.bg (P.H.); 4 R&D Center, LB Bulgaricum PLC, 14 Malashevska Str., 1225 Sofia, Bulgaria; zoltan_urshev@yahoo.com

**Keywords:** *Xanthomonas euvesicatoria*, pepper plants, biotic stress, bacteriophage, biocontrol

## Abstract

The present study was focused on the pathosystem pepper plants (*Capsicum annuum* L.)-phytopathogenic bacterium *X. euvesicatoria* (wild strain 269p)-bacteriophage BsXeu269p/3 and the possibility of bacteriophage-mediated biocontrol of the disease. Two new model systems were designed for the monitoring of the effect of the phage treatment on the infectious process in vivo. The spread of the bacteriophage and the pathogen was monitored by qPCR. A new pair of primers for phage detection via qPCR was designed, as well as probes for TaqMan qPCR. The epiphytic bacterial population and the potential bacteriolytic effect of BsXeu269p/3 in vivo was observed by SEM. An aerosol-mediated transmission model system demonstrated that treatment with BsXeu269p/3 reduced the amount of *X. euvesicatoria* on the leaf surface five-fold. The needle-pricking model system showed a significant reduction of the amount of the pathogen in infectious lesions treated with BsXeu269p/3 (av. 59.7%), compared to the untreated control. We found that the phage titer is 10-fold higher in the infection lesions but it was still discoverable even in the absence of the specific host in the leaves. This is the first report of in vivo assessment of the biocontrol potential of locally isolated phages against BS pathogen *X. euvesicatoria* in Bulgaria.

## 1. Introduction

Bacterial spot (BS) along with other bacterial diseases in crops are among the main reasons for great yield losses worldwide having an influence both on human nutrition and on the economies of the producing countries. Globally, BS strikes down two important crops (tomato and pepper) and is caused by four distinct phytopathogenic bacterial species [1]. All of them are included in the A2 list of pests recommended for regulation as quarantine pests (version 2022-09) and are put under constant quarantine control worldwide [2,3]. The long-distance dispersal of BS causative agents is favored by transfer of contaminated seeds and transplants [1]. The environmental factors that facilitate the development of BS disease are mainly rain and wind as well as high humidity and temperatures [1]. In Bulgaria, three BS pathogens (*Xanthomonas euvesicatoria* pv. *euvesicatoria, Xanthomonas vesicatoria* and *Xanthomonas hortorum* pv. *gardneri*) are widely distributed, and fighting them poses a serious challenge due to easy dispersal both in open fields and greenhouses. The population of the causative agents of BS in Bulgaria have been considerably studied over the last three decades [4,5,6,7,8,9,10,11].

The impact of BS disease on tomato and pepper has been extensively studied over the years. For example, a survey conducted in pepper fields in Turkey showed that the BS disease severity has been estimated to be between 50 and 95% [12]. In similar research from North Macedonia, the authors report that the yield losses in pepper-producing fields have been estimated to be between 10 and 20% [13]. Approximately the same yield losses (23–44%) have been reported in research aimed at clarifying the direct crop losses in pepper plants artificially infected with *Xanthomonas campestris* pv. *vesicatoria* in Israel [14].

Routinely, chemical pesticides are used for crop protection, but their use is subject to reconsideration due to the rising resistance of phytopathogenic bacteria and the negative influence they have on the environment [15,16,17,18]. Moreover, successful plant protection requires the integration of different strategies which aim to reduce the severity and to make the diseases more effectively manageable at all [19]. Therefore, the attention and hopes of the researchers are focused on the potential of a new but well-forgotten old bacterial enemy, bacteriophages, to successfully replace the usage of chemical pesticides in agriculture.

In the last few decades, the interest of researchers and business in bacteriophages as an innovative biocontrol strategy is rising [20,21]. Holtappels et al. have summarized, the published last few years data, that reveal phages’ biocontrol potential [22]. The review represents not a small number of in vitro, ex vivo and in vivo experiments, which indicates a serious increase in interest in them [22]. Bacteriophage mixtures have been reported as effective biocontrol agents against several pathogens, including pectinolytic bacteria *Dickeya solani* in potatoes [23], *Ralstonia solanacearum*, the causal agent of bacterial wilt of solanaceous plants [24] and against the bacterial soft-rot-causing agent of onion, *Pectobacterium carotovorum* subsp. *carotovorum* [25]. This might be due to the advantages that phage preparations have over chemical pesticides. Phages allow an eco-friendly and targeted application, i.e., they act and destroy only their natural bacterial host but not the beneficial soil and plant microbiota [26].

Many studies have demonstrated the application of bacteriophages against different xanthomonads [27,28,29,30]. The severity of BS (caused by *X. euvesicatoria*) has been significantly reduced in field trials after foliar treatment of artificially infected pepper plants with a combination of copper hydroxide + bacteriophage KΦ1 + acibenzolar – S – methyl (ASM) [27]. In another experiment, it has been revealed that phage X3 significantly decreases the symptoms of bacterial leaf blight in rice if applied by leaf spraying before the artificial infection with *Xanthomonas oryzae* pv. *oryzae* [28]. The ability of several *Xanthomonas perforans* phages to persist, propagate and decrease the symptoms of BS have been also evaluated, and the correlations between phage populations on leaf surfaces and disease severity have been revealed [29].

The efficacy of phage treatments is considered to depend on a few basic factors, especially under in vivo conditions: the biology of the target phytopathogenic bacterium and phages themselves, the relationship between host and pathogen in the pathosystems, the number and timing of phage application, the influence of environmental factors, etc. [22]. It is expected that leaf bacterioses might be more effectively managed by foliar phage delivery, although foliar phage application has the disadvantage of the phyllosphere being exposed to various environmental factors (UV, rains, wind, etc.) affecting phage survival and effectiveness [31,32]. On the other hand, soil-based phage delivery could be used for monitoring of soilborne phytopathogenic bacteria. Soil-based phage application could be considered as an alternative manner for phage delivery even if the target pathogen, such as *X. perforans*, *X. euvesicatoria*, *X. oryzae* and *Erwinia amylovora,* causes leaf infection due to the possible translocation of phages throughout the plant parts [33,34].

To reveal the full capacity of a particular phage isolate to effectively reduce the severity of a disease, various experiments under in vitro and in vivo conditions need to be carried out. The object of the present study is the lytic bacteriophage BsXeu269p/3, capable of infecting a broad spectrum of strains of *X. euvesicatoria*. The biocontrol potential of this phage isolate in vitro has been recently reported [35]. In previous research, we studied the BS caused by *X. euvesicatoria* in pepper and plant defense responses [36]. The aim of the present study is to establish the in vivo capacity of BsXeu269p/3 to act as a biocontrol agent against a BS pathogen, *X. euvesicatoria* strain 269p. By molecular detection of pathogens and bacteriophages, we monitored the development of the infectious process in pepper plants. We demonstrated that BsXeu269p/3 disrupts the pathogen transport between infected and healthy plants and also reduces local infection spots on the leaves. This is the first report of an in vivo assessment of the biocontrol potential of locally isolated phages against BS pathogen *X. euvesicatoria* in Bulgaria.

## 2. Results

### 2.1. Aerosol-Mediated Transmission (Model System I)

In the model system I, plants were treated with a pressurized water stream, simulating rain (see Material and Methods, Section 4.3). Our presumption was that when spraying the leaves with a strong stream of water, an aerosol, probably containing phytopathogenic bacteria, is formed and spreads in all directions. It settles on surrounding, uninfected plants and could cause disease. Thus, this laboratory experiment aimed to simulate the dispersal of the phytopathogenic bacteria through raindrops (in nature) over large area, reaching the neighboring healthy plants.

#### 2.1.1. *X. euvesicatoria* Strain 269p Was Detected in the Aerosol

**Figure 1:**
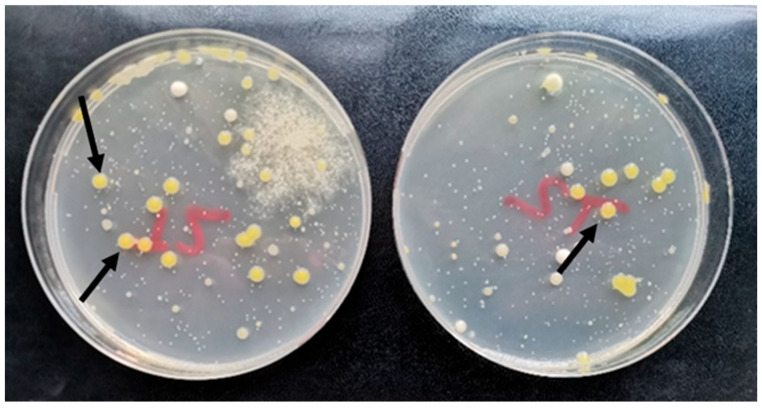
The arrows indicate the typical yellow mucoid colonies of *X. euvesicatoria* 269p on PSA observed after cultivation of droplets in MS I.

To confirm the presence of the pathogen in the droplets, we collected samples and cultivated them on petri dishes (see Section 4.4). After 72 h of cultivation, numerous colonies developed, some of which were typical for *X. euvesicatoria* 269p—yellow and mucoid (Figure 1). Species-specific PCR confirmed that the colonies were *X. euvesicatoria*. In control petri dishes, the pathogen was not found. These results clearly show that bacterial cells remained viable after the aerosolization process and could spread to neighboring healthy plants.

#### 2.1.2. BsXeu269p/3 Treatment Reduced Aerosol-Mediated Transmission (AMT) of *X. euvesicatoria* Strain 269p

After being transferred by aerosol to the leaves of neighboring, uninfected plants, the bacterium initiated a new epiphytic colonization. To assess the extent of *X. euvesicatoria* transfer to surrounding plants, as well as whether bacteriophage treatment affects this process, we developed two methodological approaches. By collecting swabs from leaf surfaces of uninfected plants, we determined the degree of infection (percentage of the pathogen-positive samples), as well as the quantity of the *X. euvesicatoria* strain 269p (Table 1). In the second method of sampling, the pathogen was isolated from the leaf tissues of peripheral, uninfected plants. In this case, the sample contained both the bacteria on the leaf surface and those that had penetrated into the leaf parenchyma (Table 2). In both approaches, DNA was isolated only from healthy leaf tissues with no visible symptoms of infection. Using qPCR analysis, we quantitatively examined the effects of phage application on horizontal bacterial transmission.

As seen in Table 1, leaf surface swabbing samples showed a 53 percent positive identification of *X. euvesicatoria* in peripheral plants. In comparison, a two-fold decrease in the percentage of positive samples (25%) was found in BsXeu269p/3-treated plants. The difference in bacterial cell counts was even more significant, as bacteriophage application reduced the pathogen quantity on the leaf surface by an average of five-fold (from 20 × 10^4^ to 4 × 10^4^ bacterial cell per mL) (Table 1). The results from the plant tissue samples showed a similar tendency (Table 2). Pooled data from the three trials showed that treatment with BsXeu269p/3 reduced the number of pathogen-positive qPCR samples. It also decreased the amount of bacterial DNA more than two-fold.

These two methodical approaches demonstrated the versatility of qPCR in assessing the degree of infection of plants with *X. euvesicatoria*. Exogenous treatment with bacteriophages reduced the amount of the bacteria on the leaf surface, thus restricting the horizontal transmission of the pathogen from diseased to healthy plants.

### 2.2. Visual Monitoring of Leaf Surface by SEM

**Figure 2:**
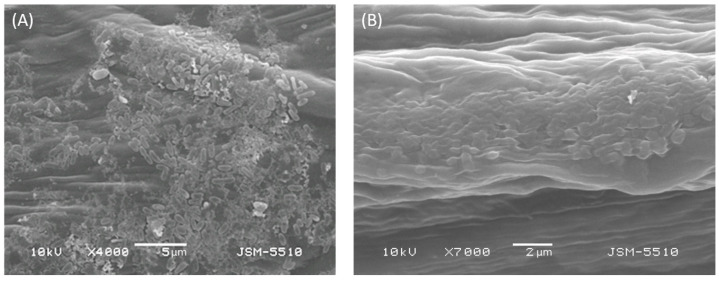
SEM micrographs showing *X. euvesicatoria* 269p epiphyte colonization (intact cells) on the ventral side of the leaf surface of *Capsicum annuum* L., cv. Sofiiska kapia, 7 dpi (**A**) and potential bacteriolytic effect of BsXeu269p/3 on the target bacterium in plant samples (**B**).

After the inoculation of pepper plants with *X. euvesicatoria* 269p, the epiphytic stage of the pathogen initiated and a dense bacterial biofilm was formed on the leaf surface (Figure 2A). As we explained above, our results from qPCR analyses showed that the treatment with specific bacteriophage BsXeu269p/3 led to a significant reduction of bacterial cells in plant tissues. We used SEM as an additional tool to try to observe the lysis and to confirm these results. Figure 2B shows the potential bacteriolytic effect of BsXeu269p/3 on *X. euvesicatoria* strain 269p cells in plant samples. An inhomogeneous plaque of partially lysed cells can be observed in the image instead of vital cells.

### 2.3. Needle Pricking (Model System II)

In model system I, we demonstrated that treatment with BsXeu269p/3 reduced the transfer of *X. euvesicatoria* from infected to healthy plants. However, this does not answer the question of whether the bacteriophage will be also effective inside the leaf parenchyma, i.e., at the endophytic stage. Therefore, in MS II, pepper leaves previously infected with the pathogenic bacterium *X. euvesicatoria* strain 269p by needle pricking were treated twice with BsXeu269p/3 (see Materials and Methods, Section 4.5).

**Figure 3:**
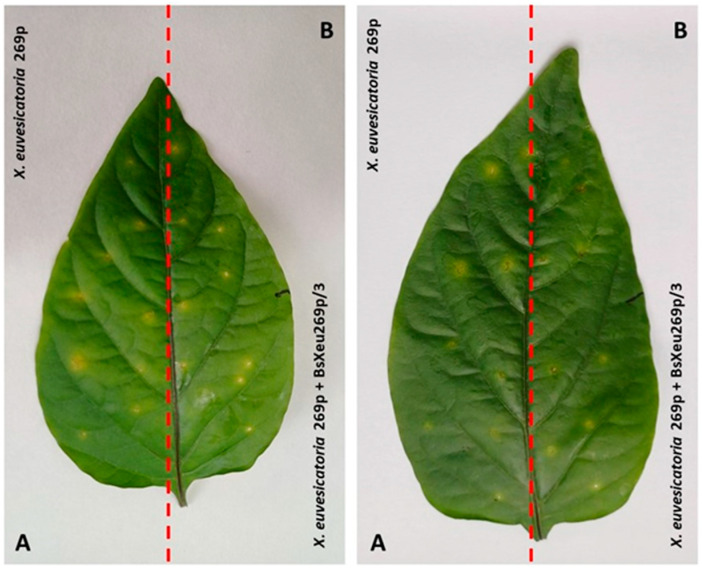
Visible chlorotic lesions of pepper plants (*Capsicum annuum* L., cv. Sofiiska kapia) inoculated by needle puncture with *X. euvesicatoria* 269p (**A**) and reduced visible symptoms in the same leaf after subsequent treatment with BsXeu269p/3 (**B**), 11 dpi, MS II.

Approximately 7 dpi (day post inoculation), single yellow-green chlorotic circular lesions on the inoculation points occurred (Figure 3A). Ten days later, the lesions necrotized and changed in color from dark brown to black in the center. Finally, the process led to the drying of the leaves and plant defoliation. As seen in Figure 3B, treatment of the leaf surface with the BsXeu269p/3 bacteriophage slightly reduced the visible lesions.

In MS II, we optimized TaqMan qPCR for phage and bacterial quantification in plant samples. To quantify the phage BsXeu269p/3 DNA, a pair of new specific primers was designed, based on the putative major capsid protein gene. Template DNA was extracted from leaf discs centered around the needle punctures.

**Figure 4:**
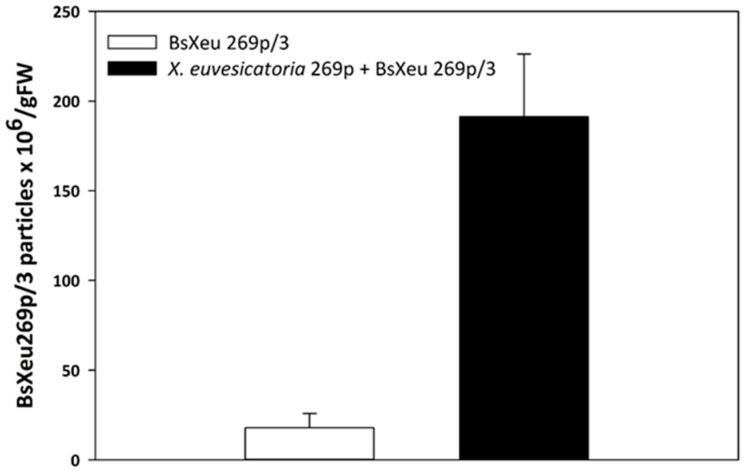
BsXeu269p/3 reproduction in leaves of pepper plants (*Capsicum annuum* L., cv. Sofiiska kapia), in the presence or absence of *X. euvesicatoria* 269p. Phage quantification was performed by TaqMan qPCR assay. The presented values are the results of three independent experiments. The data presented in the table are mean values ± SE.

The application of BsXeu269p/3 to *X. euvesicatoria* 269p-treated pepper plants reduced the amount of pathogens at the inoculation points by an average of 59.7% (see Table 3). This result confirmed that the bacteriophage could penetrate the leaf parenchyma and directly attack the target bacteria. It has been reported that the phage BsXeu269p/3 has a high specificity for *X. euvesicatoria* [35]. In the absence of *X. euvesicatoria* 269p, the phage quantity gradually decreased. Our results showed that the amount of phage in infectious lesions was more than 10-fold higher compared to the healthy part of the leaf (Figure 4).

These results confirmed in an in vivo experimental setting that the test phage could multiply in the presence of the pathogen. At the inoculation points, the phage quantity increased, while the bacterial load was reduced, and consequently the plant was less damaged by the disease.

## 3. Discussion

### 3.1. Bacteriophages as Biocontrol Agents

Nowadays, the successful control of bacterial diseases on plants requires the study, development and application of combined strategies with a strict focus on their effectiveness and environmental safety. Therefore, this inevitably leads to the replacement of traditionally used chemical pesticides which have been reported to have serious disadvantages: increasing inefficiency due to rising resistance among phytopathogenic bacteria [16,17,37] and the harm they cause to the environment and human health [15]. Application of bacteriophages as biocontrol agents in the integrated plant protection strategy is among the most intensively studied approaches [22].

An essential part of the present study is focused on the development of assays for the evaluation of the bacteriophage BsXeu269p/3 potential as a biocontrol agent against BS agent *X. euvesicatoria* under in vivo conditions. The BsXeu269p/3 is a lytic tailed phage which has been previously isolated and characterized. It has a broad host range, as it has lytic activity against a wide range of tested strains of the species *X. euvesicatoria* (n = 33). The safety status of the phage has also been proved as no lytic activity was detected against beneficial soil microorganisms (heterotrophs, nitrogen-fixing, ammonifying and denitrifying bacteria). The phage is also tolerant to a large scale of pH and possesses excellent viability at 4 °C and −20 °C for a long period of time. Moreover, this phage can persist in soil for 55 days without the presence of its natural hosts [35]. All these beneficial phage characteristics make this phage isolate suitable for in-depth analyses. Precisely for this reason, BsXeu269p/3 was chosen for conducting the experiments reported in this paper.

### 3.2. X. euvesicatoria and Its Dissemination

*Xanthomonas euvesicatoria* pv. *euvesicatoria* (former *X. euvesicatoria*) has been considered as a major causative agent of bacterial leaf spot disease in *Capsicum annuum* L. and *Solanum lycopersicum* L. crops [1]. This phytopathogenic bacteria represents a serious problem for agriculture in many countries around the world, leading to great economical losses [38]. Usually, the bacteria enter into plant tissue via natural openings and wounds and colonize the apoplast, causing lesions of the leaves, stems, and fruits [39]. The most important environmental factors that significantly favor the spread of the pathogen are wind and rain [39].

In our previous work, we studied the pathosystem pepper (*Capsicum annuum* L.)-*X. euvesicatoria* 269p (wild strain) under controlled conditions [36]. The most important outcome was that the invasion of the pathogen causes local infection and that the additional dissemination of bacteria in the healthy parts of the host is blocked, probably with the participation of an oxidative stress response. In agreement with other authors [39], we assume that natural factors, such as wind, rain and insects, are the main reason for the spread of *X. euvesicatoria.* Raindrops can simultaneously transport pathogens and, along with the mechanical injures of the leaves, provide entry points for bacteria into the leaf parenchyma. Rain provokes the simultaneous development of multiple local infectious sites, and this is probably the main reason for the massive invasion of pathogens in field conditions. The described route of pathogen spread explains why a pathogen such as *X. euvesicatoria*, which causes a local infection, can contribute to a general and significant damage to crops.

### 3.3. Bacteriolytic Effect of BsXeu269p/3 on X. euvesicatoria 269p

The infection lifecycle of the xanthomonads and *X. euvesicatoria* in particular includes two stages: epiphytic and endophytic [39]. For the development of the primary inoculum, dissemination, and survival of pathogens, the epiphytic stage is crucial. Most likely, in a natural environment, the bacterial population in the epiphytic stage is the main reservoir and source for the spread of infection. In our experiments, the epiphytic population (a dense bacterial biofilm) of *X. euvesicatoria* strain 269p formed after artificial infection of pepper plants was observed by SEM (Figure 2). The treatment with specific bacteriophage BsXeu269p/3 led to a visual change in the appearance of the bacterial biofilm on the leaf surface (Figure 2B). We compared the obtained images with similar ones reported in the literature. For example, the bacteriolytic effect of purified phage lytic enzyme P9ly on *Shigella dysenteriae* and *Staphylococcus aureus* has been observed by SEM [40]. On the basis of comparison, we tend to define the image obtained from our phage-treated leaf specimens as a potential bacteriolytic effect of BsXeu269p/3 on *X. euvesicatoria* strain 269p cells.

Two model systems of artificial infection and phage application were designed for the purposes of this study: model system I (aerosol-mediated transmission) and model system II (needle pricking). Both systems are based on foliar application of the bacteriophages. The reason for choosing this type of application is that *X. euvesicatoria* is a pathogen that causes leaf bacteriosis on tomato and pepper [1], and it is expected that in this case the contact between the phytopathogenic bacteria and the bacteriophage and the result of this contact will be most efficient. Foliar application of bacteriophages against different xanthomonads has also been explored and reported [27,28,29,30]. 

### 3.4. Model System I

The main idea of Model system I (aerosol-mediated transmission) was to establish the degree of dispersal of the phytopathogenic bacteria through simulated raindrops and the role of the bacteriophage BsXeu269p/3 in reducing this dispersal and thus, in reducing the possibility of disease development in the surrounding healthy plants. In this originally designed model system, pepper plants were artificially infected with *X. euvesicatoria* strain 269p and treated with a pressurized water stream, simulating rain. We proved that the resulting aerosol contained viable cells of *X. euvesicatoria.* Actually, the bacterial cells, released from the leaf surface by the water stream, are capable of translocating to the surrounding healthy plants. Similar simulative laboratory experiments exploring the role of wind in the dispersal of citrus canker pathogen (*Xanthomonas citri* subsp. citri) have also been reported, concluding that wind is the major environmental factor having an influence on bacterial dispersal [41].

A qualitative and quantitative assessment of the degree of transfer of the pathogen from infected to the surrounding healthy plants was made. Usually, visual assessment is used to assess the extent of plant damage by pathogens. For example, Gasic et al. [30] have reported that foliar applications of the phage KΦ1 reduced the average number of bacterial spot lesion on pepper leaves. We chose to avoid visual observation but to use qPCR instead. Molecular detection of pathogens provides sensitive, quantitative and qualitative analytical tools appropriate for monitoring plant disease development. This method allows the detection of minimal amounts of pathogen DNA in plant materials, including tissues and leaf surfaces. To prove the translocation of phytopathogenic bacteria to neighboring healthy plants and the role of bacteriophage BsXeu269p/3 in reducing the quantity of translocated bacteria to the peripheral healthy plants, we collected two types of samples: swab specimens and leaf-tissue specimens. The results obtained after qPCR analyses showed that foliar application of phage suspension by spraying significantly reduces the amount of *X. euvesicatoria* strain 269p in the surrounding plants. The qPCR results from swab specimens showed a two-fold reduction of *X. euvesicatoria*-positive samples in surrounding plants after treatment with BsXeu269p/3, as well as a five-fold reduction in the amount of bacterial DNA (Table 1). A similar but less pronounced tendency was found for DNA isolated from leaf-tissue segments (Table 2).

These results could be considered logical, as foliar treatment allows the bacteriophage to directly attack the bacteria on the leaf surface. We also could explain the great percentage of bacterial reduction in phage-treated plants with the timing of phage application. In our experiments, phage preparations were applied twice, at 2 and 8 dpi. This probably gave the bacteria the needed time to multiply on leaf surfaces and thus to become easily accessible by the phage. This step could be considered crucial for efficient phage multiplication and thus, for effective reduction of the bacterial concentration.

Several studies regarding foliar phage application can also be mentioned: phage Phi 6 has been reported to successfully reduce the titer of *Pseudomonas syringae* pv. *actinidiae* after ex vivo application in artificially infected kiwifruit leaves [42]; the bacteriophage Medea 1 was tested for effectiveness against *Pseudomonas syringae* pv. *tomato* (Pst) (the causative agent of bacterial speck on tomato) by foliar and root-drenching application and as a more effective approach, reaching four-fold reduction of the symptoms of Pst on tomato plants; (foliar application has been reported) [43]; a significant reduction of bacterial leaf blight disease symptoms in rice, caused by *Xanthomonas oryzae* pv. *oryzae* after foliar application of phage X3 under in vivo conditions, has also been reported [28]. 

In summary, for MS I, the foliar administration of BsXeu269p/3 reduces the amounts of bacteria in their main reservoir (leaf surface) and thus reduces the possibility for pathogen dispersal to new hosts. Each new cycle of bacteriophage treatment reduces the epiphytic population and lowers the opportunity for *X. euvesicatoria* to transfer with rain or insects into neighboring healthy plants. The plants that are the source of the infection may have significant damage, but the bacteriophages can block the spread of the pathogens and thus protect the entire plant population.

### 3.5. Model System II

**Figure 5:**
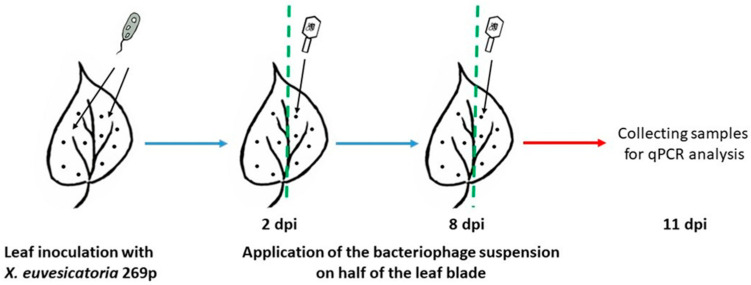
Scheme of MS II. Pepper plants (*Capsicum annuum* L., cv. Sofiiska kapia) were infected with *X. euvesicatoria* 269p (4 × 10^8^ cfu/mL) by needle-pricking method. BsXeu269p/3 treatments were made at 2 and 8 dpi. The samples for TaqMan qPCR were collected at 11 dpi.

Our MSII (needle-pricking model system) aimed to demonstrate the action of the phage BsXeu269p/3 at the point of infection and to establish the ability of BsXeu269p/3 to penetrate the leaf parenchyma, reaching the endophytic bacterial population. It was important for us that the quantification of microorganisms by qPCR be performed under strictly uniform conditions. For this reason, the samples for the two experimental variants, the pathogen alone and in combination with the bacteriophage, were collected from the two halves of the same leaf of the plant (see Figure 5). In our previous work, we found that *X. euvesicatoria* induces strictly local infection under controlled conditions, which make the lesions on leaves compact and easy to differentiate [36]. For precise quantitative measurements of pathogen and BsXeu269p/3 DNA, new primer pairs and probes for TaqMan qPCR were designed (see Material and Methods, Section 4.9). The results presented in Table 3 show that the amount of *X. euvesicatoria* DNA in infectious lesions treated with BsXeu269p/3 is reduced by an average of 59.7% compared to specimens treated only with *X. euvesicatoria* strain 269p. This demonstrates the ability of the bacteriophage to penetrate deep into the leaf parenchyma and attack its targets there. Such penetration is presumably due to the sequential attack of bacterial cells by BsXeu269p/3, resulting in a chain effect. However, inhibition of bacterial growth had little effect on the visual symptoms of infection (Figure 3). Eventually, the pathogen-affected leaves wither, and the bacteriophage treatment extends their life by only a few days. When the bacterium has penetrated the leaf tissues, the application of the phage slows down its development but cannot “cure” the affected part of the plant.

Simultaneously with the bacteria, the amount of BsXeu269p/3 in infectious lesions was also determined. Control leaf samples (from the same plant, infected with strain 269p), treated only with phage suspension, were also collected. The data presented in Figure 4 show a 10-fold increase in the BsXeu269p/3 titer in infectious lesions. These results could be considered expected, as it is known that phages are capable of multiplying only if a natural host is present (as it is in the inoculation points). However, the fact that phage DNA from the control sections was also detected, even in significantly lower concentrations, in the absence of a target bacterium, could be considered promising. Although the presence of phage DNA is only indirect evidence of the persistence of virulent phage particles, even if a small part of the BsXeu269p/3 remains viable, it means that, if accidental infection occurs, phages could serve as a reservoir of active specific antibacterial agents. The ability of this phage to maintain its viability in soil for 55 days in the absence of its specific host has been reported previously [35].

## 4. Materials and Methods

### 4.1. Microorganisms

The bacterial strain used in this study (*X. euvesicatoria* strain 269p) is part of the collection of phytopathogenic bacteria (Department of General and Industrial Microbiology, Laboratory of phytopathogenic bacteria, Faculty of Biology, Sofia University “St. Kliment Ohridski”). The strain is pathogenic to pepper plants, i.e., pepper pathotype (PT) [4]. The bacterial suspensions needed for artificial infection of pepper plants were prepared from log bacterial culture (cultivated overnight in potato sucrose agar–PSA) suspended in normal saline (0.9% NaCl) until it reached 10^8^ cfu/mL (1.3 MacFarland units). The bacteriophage BsXeu269p/3 used in this study was previously isolated and characterized [35]. For the purposes of this study, it was propagated in 50–100 mL Luria Bertani broth (Yeast extract–5 g/L, Tryptone–10 g/L, NaCl–10 g/L, pH 7–7.2) along with its natural host (*X. euvesicatoria* strain 269p) at MOI–1. For optimal phage adsorption to bacterial cells, 100 mM CaCl_2_ was added in the cultivation media, and the cultivation was carried out in an orbital shaker (RSLAB-7PRO) at 220 rpm, overnight (16 h). Then, the phage lysate was filtered through a bacterial filter (Minisart^®^ Syringe Filter 28 mm, pore size 0.22 μm, Sartorius, Gottingen, Germany) for removing any live bacterial cells and stored in sterile containers at 4 °C for further analyses. Phage titers were counted via a spot testing assay [44].

### 4.2. Plant Material

Pepper plants (*Capsicum annuum* L. cv. Sofiiska kapia) were grown in a climatic chamber under controlled conditions (26 ± 2 °C temperature, 16/8 h day/night photoperiod, light intensity 70–90 µmol/m^2^/s, and 60–70% relative air humidity) in pots filled with a commercial soil mix (EKO Durpeta, Šepeta, Lithuania).

### 4.3. Model System I (Aerosol-Mediated Transmission)

The model system I (MS I) was designed to investigate the horizontal transmission of the bacterium between neighboring plants and the ability of the specific bacteriophage (BsXeu269p/3) to prevents its spread. 

**Figure 6:**
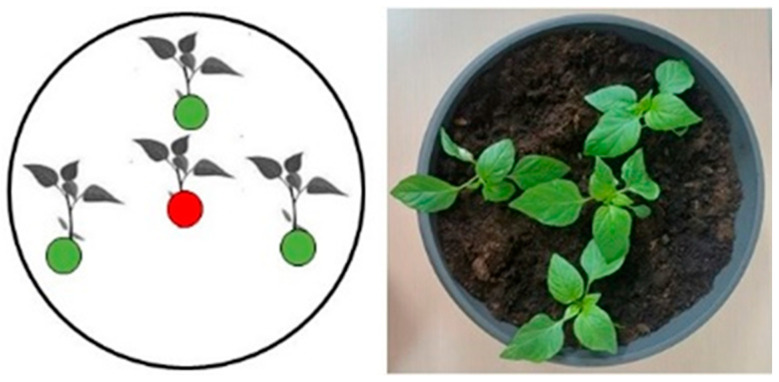
Schematic model of the experimental pots used in MSI. Each pot contains 4 pepper plants (*Capsicum annuum*, L. cv. Sofiiska kapia).

In large pots with a diameter of 40 cm, we planted 4 plants per pot: one in the center and the rest equally spaced (15 cm) around it (at 120° from each other) (Figure 6). The plants in the center (35-day-old seedlings) were inoculated by rubbing a bacterial suspension (10^8^ cfu/mL) into their leaves. The pots were covered with plastic bags and left in the dark for 24 h. It is well known that rain enhances phytopathogen transmission. Thus, in our model system, to simulate rain conditions, the plants were sprayed (using a spraying bottle) with a pressurized stream of water. 

Two days post inoculation, half of the pots were sprayed with bacteriophage suspension (10^9^ pfu/mL), covered with plastic bags, and left in the dark for 4 h. The other pots were sprayed with distilled water only and were used as controls. 

At 6 dpi, all pots were sprayed with pressurized distilled water from around 20 cm distance 15 times using a spraying bottle. Approximately 20 mL of water was used for one pot. The main target was the infected plant in the middle of the pot. 

**Figure 7:**
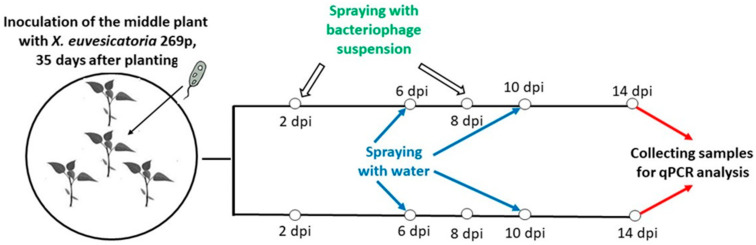
Study model of the MS I. The middle plants (*Capsicum annuum* L., cv. Sofiiska kapia) were infected with *X. euvesicatoria* 269p (10^8^ cfu/mL). The bacteriophage suspension (10^9^ cfu/mL) was sprayed on half of the experimental pots at 2 and 8 dpi. All experimental groups of plants were sprayed with water twice, at 6 and 10 dpi. A sample collection was made at 14 dpi. A minimum of three separate pots were used for one experimental group.

Re-treatment with bacteriophage suspension was carried out at 8 dpi, and the plants were again placed for 4 h in the dark. At 10 dpi, the plants were sprayed once again with pressurized water, simulating rain. Plant samples for qPCR analysis were collected from the peripheral plants at 14 dpi (Figure 7). Personal protective equipment was used during the treatment. All experiments were conducted in triplicates. 

Swabs were collected from the abaxial leaf surface of peripheral plants that were not infected directly. Samples were taken from 2 leaves per plant, from a surface with an area of approx. 10 cm^2^.

Leaf material of peripheral plants was also collected. Each sample consisted of 3 leaf discs (about 40 mg) with a diameter of 8 mm taken from a leaf without visible symptoms. Each disc was from a different leaf. Two samples per plant were collected.

### 4.4. Detection of X. euvesicatoria in the Aerosol

To detect the presence/absence of *X. euvesicatoria* strain 269p in the aerosol (simulating rain), samples were collected in petri dishes containing PSA. Before spraying, the petri dishes were placed, opened, at the level of the top of the plants so that the aerosol could settle unhindered on the agar surface. Direct spraying onto the agar surface was avoided. Control petri dishes were placed, opened, in the growth chamber for 15 min until the air above settled on the agar surface. Then, all petri dishes (containing samples and controls) were closed and incubated at 28 °C for 72 h. The experiments were carried out in duplicates. After that period, the resulting typical yellow, mucoid colonies were isolated and purified via two consecutive cultivations on PSA. Species-specific PCR analysis was carried out to prove/deny their belonging to the species *X. euvesicatoria* [45].

### 4.5. Model System II (Needle Pricking)

This model system was designed to evaluate the direct impact of bacteriophage BsXeu269p/3 on its natural phytopathogenic host, *X. euvesicatoria* strain 269p/3, and the interactions between both microorganisms on leaf tissues.

Leaves of 35-day-old pepper plants were punctured with a sterile needle dipped in a concentrated *X. euvesicatoria* 269p suspension (4 × 10^8^ cfu/mL in 0.9% NaCl). The entire leaf blade was inoculated by 5 to 8 punctures on either side of the central vein, depending on the size of the leaf (Figure 5). Inoculated plants were covered with plastic bags and were kept in the dark for 24 h. Then, a BsXeu269p/3 phage suspension (10^9^ pfu/mL) was applied twice (at 2 and 8 dpi) to one half of the leaf blade with a soft brush. After bacteriophage application, plants were kept in the dark for 4 h. Samples were collected at 11 dpi (see Figure 5). Each sample (about 40 mg) consisted of 3 leaf discs of 8 mm diameter, taken from one leaf. In the center of the disk was the needle puncture. 

### 4.6. Scanning Electron Microscopy (SEM)

Two types of probes were prepared: 1, from leaves infected with *X. euvesicatoria* strain 269p, and 2, from leaves infected with *X. euvesicatoria* 269p and subsequently treated with BsXeu269p/3. The preparation of the specimens was carried out according to a procedure described by Ganeva et al. [46]. Air-dried 0.5 cm^2^ segments from leaf surfaces of the middle part of the leaf lamina were attached to aluminum specimen stubs by double-sided carbon tape. After being sputter-coated with gold (Jeol JFC-1200 fine coater, Tokyo, Japan), the specimens were observed by scanning electron microscope Jeol JSM-5510.

### 4.7. DNA Extraction

#### 4.7.1. From Plants

The leaf material samples collected in MS I and MS II (as described above) were homogenized with 500 µL of 0.5 M NaOH containing 0.5% (*w*/*v*) polyvinylpyrrolidone (PVP). Then, 500 µL of sterile water was added and the homogenates were left for 15 min at room temperature [45]. After centrifugation (at 10,000× *g* for 10 min), the supernatants were 50-fold diluted in sterile 0.2 M Tris/HCl buffer, pH 8 for long-term storage at −20 °C.

#### 4.7.2. From Swabs

DNA extraction from swabs was carried out with One-Step DNA/RNA Extraction Buffer 10×, CHAI, according to the manufacturer’s instructions.

### 4.8. Real Time PCR

Real time PCR (qPCR) analyses were performed with iTaq Universal SYBR Green kit (Bio-Rad) according to the manufacturer’s instructions. Xeu 2.4/Xeu 2.5 primers were used [45]. All amplification reactions were carried out in PikoReal 96 with an initial step at 95 °C for 3 min, followed by 45 cycles of 95 °C for 10 s, 58 °C for 25 s and finally 60 °C for 30 s. To evaluate the number of bacteria by qPCR-based assay, we used a standard curve prepared with a known concentration of *X. euvesicatoria* 269p suspension.

### 4.9. TaqMan qPCR Assay

The primers and probes used in our study were designed based on a *X. euvesicatoria*-specific 1600 bp sequence, reported by Moretti et al. [45], and the sequence of the putative major capsid protein gene in BsXeu269p/3 (GenBank acc. no. ON996340, [35]) using the Primer3plus web interface (www.primer3plus.com, accessed on 14 January 2023) for Primer 3 [47]. The primer sequences are listed in Table 4.

TaqMan qPCR assays were run in 16 µL reactions using the SsoAdvanced™ Universal Probes Supermix Bio Rad, Hercules, CA, USA) on an PikoReal 96 Real-Time thermal cycler (ThermoScientific, Waltham, MA, USA), with an initial denaturation at 95 °C for 2 min, followed by 45 two-step cycles of denaturation at 95 °C for 15 s and annealing/elongation at 60 °C for 20 s. Quantitative estimations of the targets were performed based on standard curves obtained with known concentrations of BsXeu269p/3 and *X. euvesicatoria* 269p suspensions. All reactions were performed in 3 replicates.

## 5. Conclusions

In the end, we can draw some conclusions. The presence of *X. euvesicatoria* strain 269p in the aerosol and the proof that the bacterium was transferred to neighboring plants (MSI) are in support of the reported ways (especially rain) of dissemination of this pathogen. The bacterial population on the leaf surface is the primary reservoir for spreading the infection. The foliar treatment with BsXeu269p/3, resulting in the attack on the epiphytic population of the pathogen, reduced the amounts of viable cells capable of transfer to neighboring healthy plants, thus representing an effective bacteriophage-mediated control strategy. Additional applications with the bacteriophage enhanced its effect. qPCR detection of the pathogen from swab specimens collected from the surface of the leaves is a suitable choice for quantitative assessment of plant infection. Importantly, it is a relatively fast, easy-to-implement and a cost-saving protocol. Finally, treatment with the bacteriophage BsXeu269p/3 cannot counteract damage to an already infected plant tissue, but by destroying the *X. euvesicatoria* leaf population it is able to effectively limit the spread of the pathogen in the plant population.

**Recommendations and future perspectives**: The implementation of phage-based biopesticides in an integrated plant protection strategy for controlling bacterial diseases on crops is crucial. We strongly believe that bacteriophages can prove to be an important arsenal in the fight against resistant bacteria. Moreover, their targeted action is essential for preserving the accompanying microflora, whether applied to plants, humans or animals. Although no bacteriophage–based biopesticide has yet been officially registered in Europe by the EFSA [22], studies reporting phage potential in combating bacterial diseases at all should be stimulated.

## Figures and Tables

**Table 1:** Effect of BsXeu269p/3 application on aerosol-mediated transmission of *X. euvesicatoria* 269p in pepper plants (*Capsicum annuum* L., cv. Sofiiska kapia), MS I. The amount of *X. euvesicatoria* 269p was determined by qPCR in swab samples from the abaxial surface of asymptomatic leaves of peripheral plants. Results are shown as a percentage of positive identification of *X. euvesicatoria* 269p and as the number of bacterial cells per milliliter. The qPCR data presented in the table are mean values from three independent experiments ± SE.

	Percentage of Positive Identification on qPCR	Total Number of Probes	Bacterial Cell ×10^4^ per mL
*X. euvesicatoria* 269p	53	46	20.4 ± 5.1
*X. euvesicatoria* 269p + BsXeu269p/3	25	57	4.3 ± 0.8

**Table 2:** Effect of BsXeu269p/3 application on the aerosol-mediated transmission of *X. euvesicatoria* 269p in pepper plants (*Capsicum annuum* L., cv. Sofiiska kapia), MS I. The quantity of *X. euvesicatoria* 269p was determined by qPCR in samples of asymptomatic leaf tissue from peripheral plants. Results are shown as percent positive identification of *X. euvesicatoria* 269p and as a number of bacterial cells per gram fresh weight. The values of the three independent experiments, each consisting of different number of probes, are presented separately. The data presented in the table are mean values ± SE.

		*X. euvesicatoria* 269p	*X. euvesicatoria* 269p + BsXeu269p/3
1	Percentage of positive identification on qPCR	61.1	54.2
	Total number of probes	27	32
	Bacterial cell × 10^6^/gFW	13.2 ± 1.6	10.3 ± 1.3
2	Percentage of positive identification on qPCR	42.9	31.6
	Total number of probes	35	38
	Bacterial cell × 10^6^/gFW	24.7 ± 2	8.2 ± 1.5
3	Percentage of positive identification on qPCR	35.1	30
	Total number of probes	37	50
	Bacterial cell × 10^6^/gFW	20.1 ± 1.6	6.6 ± 0.9
Av	Percentage of positive identification on qPCR	46.4	38.6
	Total number of probes	99	120
	Bacterial cell × 10^6^/gFW	19.4 ± 1.6	8.4 ± 1.6

**Table 3:** Effect of BsXeu269p/3 application on the development of the infectious process induced by *X. euvesicatoria* 269p in pepper plants (*Capsicum annuum* L., cv. Sofiiska kapia) MS II. The amount of *X. euvesicatoria* 269p was determined by TaqMan qPCR assay in leaf samples from the lesions formed around the needle puncture. The presented values are the results of three independent experiments. The data presented in the table are mean values ± SE.

Bacterial Cell × 10^7^/gFW			Percentage of the Reduction of Bacterial Cells after BsXeu269p/3 Treatment
	*X. euvesicatoria* 269p	*X. euvesicatoria* 269p + BsXeu269p/3	
1	1581.3 ± 233.2	765.2 ± 54.5	51.6
2	255 ± 63.6	96.8 ± 28	62
3	986.7 ± 210.6	325.2 ± 90.6	66.9
Av	982.5 ± 155.9	395.7 ± 92.7	59.7

**Table 4:** List of primers used in this study.

Primers	5′-Oligo Seq-3′	Target Microorganisms	References
Xeu 2.4	CTGGGAAACTCATTCGCAGT	*X. euvesicatoria* 269p	[45]
Xeu 2.4 mod	GTTTATTGCCGGCTATCTAATCC	*X. euvesicatoria* 269p	This study
Xeu 2.5	TTGTGGCGCTCTTATTTCCT	*X. euvesicatoria* 269p	[45]
Xeu 2.4 probe	FAM-TCGGTGTTCCCTGCGACAC-TAMRA	*X. euvesicatoria* 269p	This study
YK-MjCPF1	CCAGTACGCCGTGTTCATCA	BsXeu269p/3	This study
YK-MjCPR1	GTCGTTCATCAGGCCGTAGT	BsXeu269p/3	This study
YK-MjCPF1/R1	HEX-CGGCATCGACTGGGCTGCGCGCC-BHQ1	BsXeu269p/3	This study

## Data Availability

The data presented in this study are available in the article.
